# Long-Term Outcomes of Elderly Brain Arteriovenous Malformations After Different Management Modalities: A Multicenter Retrospective Study

**DOI:** 10.3389/fnagi.2021.609588

**Published:** 2021-02-18

**Authors:** Yu Chen, Debin Yan, Zhipeng Li, Li Ma, Yahui Zhao, Hao Wang, Xun Ye, Xiangyu Meng, Hengwei Jin, Youxiang Li, Dezhi Gao, Shibin Sun, Ali Liu, Shuo Wang, Xiaolin Chen, Yuanli Zhao

**Affiliations:** ^1^Department of Neurosurgery, Beijing Tiantan Hospital, Capital Medical University, Beijing, China; ^2^Department of Neurosurgery, Peking University International Hospital, Peking University, Beijing, China; ^3^Department of Interventional Neuroradiology, Beijing Tiantan Hospital, Capital Medical University, Beijing, China; ^4^Department of Gamma-Knife Center, Beijing Tiantan Hospital, Capital Medical University, Beijing, China

**Keywords:** arteriovenous malformation, elderly, outcomes, conservation, intervention

## Abstract

**Background:** More and more elderly patients are being diagnosed with arteriovenous malformation (AVM) in this global aging society, while the treatment strategy remains controversial among these aging population. This study aimed to clarify the long-term outcomes of elderly AVMs after different management modalities.

**Methods:** The authors retrospectively reviewed 71 elderly AVMs (>60 years) in two tertiary neurosurgery centers between 2011 and 2019. Patients were divided into four groups: conservation, microsurgery, embolization, and stereotactic radiosurgery (SRS). The perioperative complications, short-term and long-term neurological outcomes, obliteration rates, annualized rupture risk, and mortality rates were compared among different management modalities in the ruptured and unruptured subgroups. Kaplan-Meier survival analysis was employed to compare the death-free survival rates among different management modalities. Logistic regression analyses were conducted to calculate the odds ratios (ORs) and 95% confidence intervals (CI) for predictors of long-term unfavorable outcomes (mRS > 2).

**Results:** A total of 71 elderly AVMs were followed up for an average of 4.2 ± 2.3 years. Fifty-four (76.1%) presented with hemorrhage, and the preoperative annualized rupture risk was 9.4%. Among these patients, 21 cases (29.6%) received conservative treatment, 30 (42.3%) underwent microsurgical resection, 13 (18.3%) received embolization, and 7 (9.9%) underwent SRS. In the prognostic comparison, the short-term and long-term neurological outcomes were similar between conservation and intervention both in the ruptured and unruptured subgroups (ruptured: *p* = 0.096, *p* = 0.904, respectively; unruptured: *p* = 0.568, *p* = 0.306, respectively). In the ruptured subgroup, the intervention cannot reduce long-term mortality (*p* = 0.654) despite the significant reduction of subsequent hemorrhage than conservation (*p* = 0.014), and the main cause of death in the intervention group was treatment-related complications (five of seven, 71.4%). In the logistic regression analysis, higher admission mRS score (OR 3.070, 95% CI 1.559–6.043, *p* = 0.001) was the independent predictor of long-term unfavorable outcomes (mRS>2) in the intervention group, while complete obliteration (OR 0.146, 95% CI 0.026–0.828, *p* = 0.030) was the protective factor.

**Conclusions:** The long-term outcomes of elderly AVMs after different management modalities were similar. Intervention for unruptured elderly AVMs was not recommended. For those ruptured, we should carefully weigh the risk of subsequent hemorrhage and treatment-related complications. Besides, complete obliteration should be pursued once the intervention was initiated.

**Clinical Trial Registration:** http://www.clinicaltrials.gov. Unique identifier: NCT04136860

## Introduction

Brain arteriovenous malformations (AVMs) were described as cerebrovascular abnormalities with fistulous connections between arteries and veins without normal intervening capillary beds (Crawford et al., 1986; Solomon and Connolly, 2017; Goldberg et al., 2018). Most AVMs were diagnosed in the fourth and fifth decade of life (Perret and Nishioka, 1966), and elderly AVMs were relatively uncommon in clinical practice. Over the past three decades, neurosurgeons have not yet reached a consensus on whether or not to intervene in these patients. Initially, several studies suggested that the risk of rupture decreases as a person reaches middle age, and these lesions are relatively benign in elderly patients (Luessenhop and Rosa, 1984; Heros and Tu, 1987). However, Harbaugh et al. suggested the opposite (Harbaugh and Harbaugh, 1994). Several subsequent studies reported that 35.7–65.6% of elderly AVMs presented with hemorrhage, and they recommended microsurgical resection or stereotactic radiosurgical surgery (SRS) for carefully selected patients (Hashimoto et al., 2004; Nagata et al., 2006; Pabaney et al., 2016; Burkhardt et al., 2018; Chen et al., 2018). However, the previous studies only included a single treatment strategy for analysis and did not compare the long-term outcomes of different management modalities.

As life expectancy continues to increase in this global aging society, more elderly AVMs are being diagnosed. We must clarify the long-term outcomes of different management modalities for these patients. The present study retrospectively reviewed 71 elderly AVMs from our multi-center retrospective database of 2861 AVMs to specify the natural history and long-term outcomes after different management modalities.

## Materials and Methods

### Study Design and Participants

We retrospectively reviewed all elderly brain AVM patients (>60 years) admitted to Beijing Tiantan Hospital and Peking University International Hospital between April 2011 and July 2019. The inclusion criteria were as follows: (1) Diagnosed with AVM by digital subtraction angiography (DSA) and/or magnetic resonance imaging; (2) The patient's age was 60 years or older on admission. Exclusion criteria were: (1) Patient's concomitant diagnosis of hereditary hemorrhagic telangiectasia; (2) Patients missing critical baseline information or those lost to follow-up; and (3) Patients who received intervention before admission. The study was carried out according to the Helsinki Declaration guideline and was approved by the ethics committees of these two hospitals.

**Figure 1:**
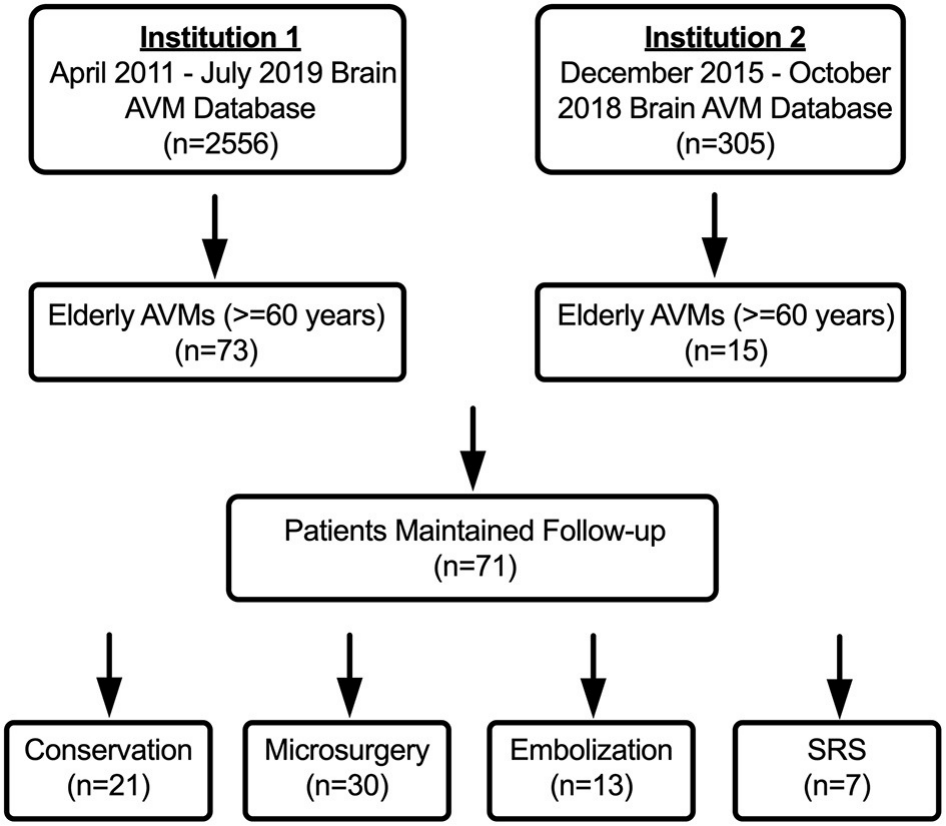
The flow diagram of patient screening. Institution 1: Department of Neurosurgery, Beijing Tiantan Hospital, Beijing, China; Institution 2: Department of Neurosurgery, Peking University International Hospital, Beijing, China.

A total of 88 elderly AVMs met the inclusion criteria from our multi-center retrospective database of 2861 AVMs, of which 17 patients were lost to follow-up. The baseline characteristics were consistent between patients who were lost to follow-up and patients who maintained followed up (Supplementary Material 1). Finally, the remaining 71 elderly AVM patients were included in the statistical analysis (Figure 1).

### Data Collection and Variable Definition

The baseline clinical characteristics included age on admission, sex, onset manifestation (hemorrhage, seizure, neurofunctional deficits, and others), and neurological status. The hemorrhagic presentation was defined as hemorrhage that could be ascribed to AVM rupture. The definition of eloquent area and deep venous drainage was consistent with the evaluation criteria in the Spetzler-Martin (SM) Grading system (Spetzler and Martin, 1986). Treatment modalities included conservation, microsurgical resection, embolization, and SRS. The rupture risk was represented by annualized rupture risk, and we defined the observational interval of natural history as the first diagnosis of AVM to admission (Chen et al., 2020). In-hospital complications were defined as intracranial hemorrhage, epilepsy, new-onset neurofunctional deficits, wound infection, intracranial infection, lung infection, major adverse cardiac events (MACEs, with the occurrence of an arrhythmia, myocardial infarction, acute heart failure, and cardiac arrest), deep venous thrombosis (DVT), and electrolyte disturbance. The neurofunctional status was evaluated by the modified Rankin Scale (mRS), and mRS > 2 was considered as neurological disabilities. Subsequent hemorrhage was defined as any hemorrhage attributable to AVM rupture during the follow-up.

Follow-up was conducted at the first 3–6 months and annually by clinical visits and telephone interviews. Two neurosurgeons with at least 5 years' experience in clinical practice evaluated all the clinical parameters. All the images were independently interpreted by at least two radiologists who worked more than 5 years in our institute's radiology center. Researchers who performed follow-up assessments were blinded to treatment modalities.

### Statistical Analysis

Categorical variables are presented as counts (with percentages); continuous variables are presented as the mean ± standard deviations (SD). Patients were divided into four groups based on different management modalities. In the comparison of the baseline characteristics, perioperative complications, short-term and long-term outcomes among different management modalities, the Pearson chi-square test, Fisher exact test, or Kruskal-Wallis ANOVA test were used to compare categorical variables as appropriate, and the two-tailed *t*-test or one-way ANOVA test were employed to compare continuous variables (normal distribution variables). Wilcoxon rank-sum test was applied to compare non-normal distribution continuous variables. Poisson rate test was used to compare the differences in annualized rupture risk. Bonferroni correction was adopted in the adjusted *post-hoc* analysis to avoid Type I errors in subgroup analyses. The subgroup analyses were conducted to compare the outcomes of different management modalities in the ruptured and unruptured elderly AVMs. Kaplan-Meier survival analysis was employed to compare the death-free survival rates (all causes, AVM and treatment-related) among different management modalities (only included patients in the first three-quarters of the follow-up duration). Univariate and multivariate logistic regression analyses were used to calculated odds ratios (ORs) and 95% confidence intervals (CI) for predictors of long-term neurological disabilities or death (mRS > 2). A forward stepwise regression procedure was adopted in the multivariable model. *P*-value < 0.05 was considered to be statistically significant. Statistical analysis was performed using SPSS (version 25.0, IBM, New York, USA).

## Results

### Baseline Characteristics

A total of 71 elderly AVMs were included according to the inclusion and exclusion criteria. Among them, 21 patients (29.6%) took conservative management and 50 patients (70.4%) received intervention, including 30 patients (42.3%) undergoing microsurgery, 13 patients (18.3%) embolization, and seven patients (9.9%) SRS. The average age was 64.7 ± 3.5 years (range, 60.0–75.2 years), and 34 patients (47.9%) were older than 65 years (Table 1). There were 54 patients (76.1%) presenting with hemorrhage. The presentation, SM grade, and imaging features between any two treatment strategies showed no significant difference after adjusted *post-hoc* Bonferroni correction analysis. From the first diagnosis to admission, 11 patients occurred 14 rupture events during the cumulative observational duration of 148.9 patient-years, translating to the natural annualized rupture risk of 9.4%. The mean admission mRS score was 1.4 ± 1.3, and the microsurgery group had a significant higher mRS score than conservation and SRS groups after adjusted *post-hoc* Bonferroni correction analysis (*p* = 0.021, *p* = 0.013, respectively), which may be caused by the fact that emergency patients (with higher mRS scores) were more likely to receive microsurgical resection.

**Table 1:** Baseline characteristics of the included elderly bAVMs.

**Characteristics**	**Total (*n* = 71)**	**Conservation (*n* = 21)**	**Microsurgery (*n* = 30)**	**Embolization (*n* = 13)**	**SRS (*n* = 7)**	***p*-value**
Sex (male)	52 (73.2)	13 (61.9)	20 (66.7)	11 (84.6)	6 (85.7)	0.412
Age (years)	64.7 ± 3.5	64.5 ± 4.0	65.8 ± 3.5	64.1 ± 2.2	62.2 ± 1.9	0.060
Age (>65 years)	34 (47.9)	9 (42.9)	19 (63.3)	5 (38.5)	1 (14.3)	0.068
Onset manifestation (primary)						
Hemorrhage	54 (76.1)	13 (61.9)	28 (93.3)	9 (69.2)	4 (57.1)	0.019^*^
Seizure	4 (5.6)	0 (0.0)	2 (6.7)	1 (7.7)	1 (14.3)	0.349
Neurofunctional deficit	4 (5.6)	2 (9.5)	0 (0.0)	1 (7.7)	1 (14.3)	0.189
Others	9 (12.7)	6 (28.6)	0 (0.0)	2 (15.4)	1 (14.3)	0.008^*^
No. of hemorrhagic events between diagnosis and treatment	14	2	6	4	2	
Annualized rupture risk	9.4%	6.3%	13.6%	8.4%	7.9%	
Admission mRS score	1.4 ± 1.3	1.1 ± 0.9	2.0 ± 1.4	1.1 ± 0.6	0.6 ± 0.5	0.002^*^
Size (cm)	2.9 ± 1.5	3.0 ± 1.5	3.2 ± 1.4	2.8 ± 1.8	1.7 ± 0.5	0.021^*^
Eloquent area	41 (57.7)	13 (61.9)	14 (46.7)	10 (76.9)	4 (57.1)	0.290
Supratentorial location	51 (71.8)	15 (71.4)	24 (80.0)	8 (61.5)	4 (57.1)	0.496
Deep venous drainage	31 (43.7)	11 (52.3)	10 (33.3)	7 (53.8)	3 (42.9)	0.471
SM grade						0.620
I	15 (21.1)	3 (14.3)	8 (26.7)	2 (15.4)	2 (28.6)	
II	22 (31.0)	6 (28.6)	10 (33.3)	3 (23.1)	3 (42.9)	
III	24 (33.8)	8 (38.1)	7 (23.3)	7 (53.8)	2 (28.6)	
IV	7 (9.9)	3 (14.3)	4 (13.3)	0 (0.0)	0 (0.0)	
V	3 (4.2)	1 (4.8)	1 (3.3)	1 (7.7)	0 (0.0)	
Follow-up duration (years)	4.2 ± 2.3	4.3 ± 2.3	4.3 ± 2.6	3.5 ± 1.9	4.5 ± 1.3	0.695
Angioarchitecture characteristics (DSA available, *n* = 53)	*n* = 53	*n* = 20	*n* = 20	*n* = 12	*n* = 1	
Drainage venous stenosis	27 (50.9)	10 (50.0)	13 (65.0)	4 (33.3)	0 (0.0)	0.207
Long venous drainage	32 (60.4)	12 (60.0)	16 (80.0)	4 (33.3)	0 (0.0)	0.346
Deep perforating arteries	17 (32.1)	8 (40.0)	3 (15.0)	5 (41.7)	1 (0.0)	0.053
Diffuse nidus	21 (39.6)	7 (35.0)	7 (35.0)	6 (50.0)	1 (100.0)	0.360
Aneurysms (flow-related)	11 (20.8)	2 (10.0)	7 (35.0)	2 (16.7)	0 (0.0)	0.235

*AVM, Arteriovenous Malformation; DSA, Digital Subtraction Angiography; mRS, modified Rankin Scale; SM grade, Spetzler-Martin grade; SRS, Stereotactic Radiosurgery*.

*Values are expressed as number of cases (%) or mean ± standard deviation, unless otherwise indicated*.

* *Statistical significance (p < 0.05)*.

Most of the elderly patients were classified as SM grade I-III (61 cases, 85.9%). There was no significant difference in the SM grade among different management modalities after adjusted *post-hoc* Bonferroni correction analysis. Of the 53 DSA-available elderly AVMs, the angiographic characteristics were similar among these four management modalities.

### Clinical Outcomes

The incidence of perioperative complications of microsurgical resection was significantly higher than that of embolization and SRS (*p* = 0.007, *p* < 0.001, respectively) (Table 2). In the microsurgery group, 13.3% patients occurred intracranial hemorrhage, 23.3% intracranial infection, 6.7% MACEs, and 20.0% DVT. In the embolization group, two patients (15.4%) experienced intraoperative hemorrhage, which led to serious perioperative complications, such as new-onset neurofunctional deficits, lung infection, DVT, and electrolyte disturbance. The discharge mRS scores were similar between different management modalities after adjusting the *post-hoc* Bonferroni correction analysis (*p* > 0.05).

**Table 2:** Perioperative complications and long-term outcomes among different treatment modalities in the elderly AVMs.

**Characteristics**	**Conservation (*n* = 21)**	**Microsurgery (*n* = 30)**	**Embolization (*n* = 13)**	**SRS (*n* = 7)**	***p*-value**
Perioperative complications	NA	18 (60.0)	2 (15.4)	0 (0.0)	<0.001^*^
Intracranial hemorrhage	NA	4 (13.3)	2 (15.4)	0 (0.0)	0.374
Epilepsy	NA	1 (3.3)	1 (7.7)	0 (0.0)	0.614
New-onset neurofunctional deficit	NA	6 (20.0)	2 (15.4)	0 (0.0)	0.249
Wound infection	NA	2 (6.7)	1 (7.7)	0 (0.0)	0.622
Intracranial infection	NA	7 (23.3)	0 (0.0)	0 (0.0)	0.019^*^
Lung infection	NA	5 (16.7)	2 (15.4)	0 (0.0)	0.317
MACEs	NA	2 (6.7)	1 (7.7)	0 (0.0)	0.622
DVT	NA	6 (20.0)	2 (15.4)	0 (0.0)	0.249
Electrolyte disturbance	NA	7 (23.3)	2 (15.4)	0 (0.0)	0.184
Discharge mRS	0.9 ± 0.7	1.7 ± 1.4	1.7 ± 1.7	0.6 ± 0.5	0.031^*^
Follow-up duration (years)	4.3 ± 2.3	4.3 ± 2.6	3.5 ± 1.9	4.5 ± 1.3	0.695
Obliteration	0 (0.0)	28 (93.3)	1 (7.7)	3 (42.9)	<0.001^*^
Long-term mRS score	1.7 ± 2.3	2.1 ± 2.2	2.2 ± 2.3	1.1 ± 2.3	0.721
Neurological disability (mRS > 2)	4 (19.0)	11 (36.7)	4 (30.8)	1 (14.3)	0.431
Worsened mRS	4 (19.0)	7 (23.3)	5 (38.5)	2 (28.6)	0.648
No. of subsequent hemorrhage	4	1	0	0	0.073
Annualized rupture risk^a^	4.4%	0.8%	0.0%	0.0%	
Death	4 (19.0)	4 (13.3)	3 (23.1)	1 (14.3)	0.870
Annualized mortality (all causes)	4.4%	3.1%	6.6%	3.2%	
Annualized mortality (AVM-related)	3.3%	0.8%	0.0%	3.2%	
Annualized mortality (treatment-related)	NA	2.3%	4.4%	0.0%	
Annualized mortality (other causes)	1.1%	0.0%	2.2%	0.0%	

*AVM, Arteriovenous Malformation; DVT, Deep Vein Thrombosis; MACE, Major Adverse Cardiac Events; mRS, modified Rankin Scale; SM grade, Spetzler-Martin grade; SRS, Stereotactic Radiosurgery*.

*Values are expressed as number of cases (%) or mean ± standard deviation, unless otherwise indicated*.

a *Poisson rate test of annualized rupture risk for conservation and intervention during follow-up is significant (p = 0.040)*.

* *Statistical significance (p < 0.05)*.

**Figure 2:**
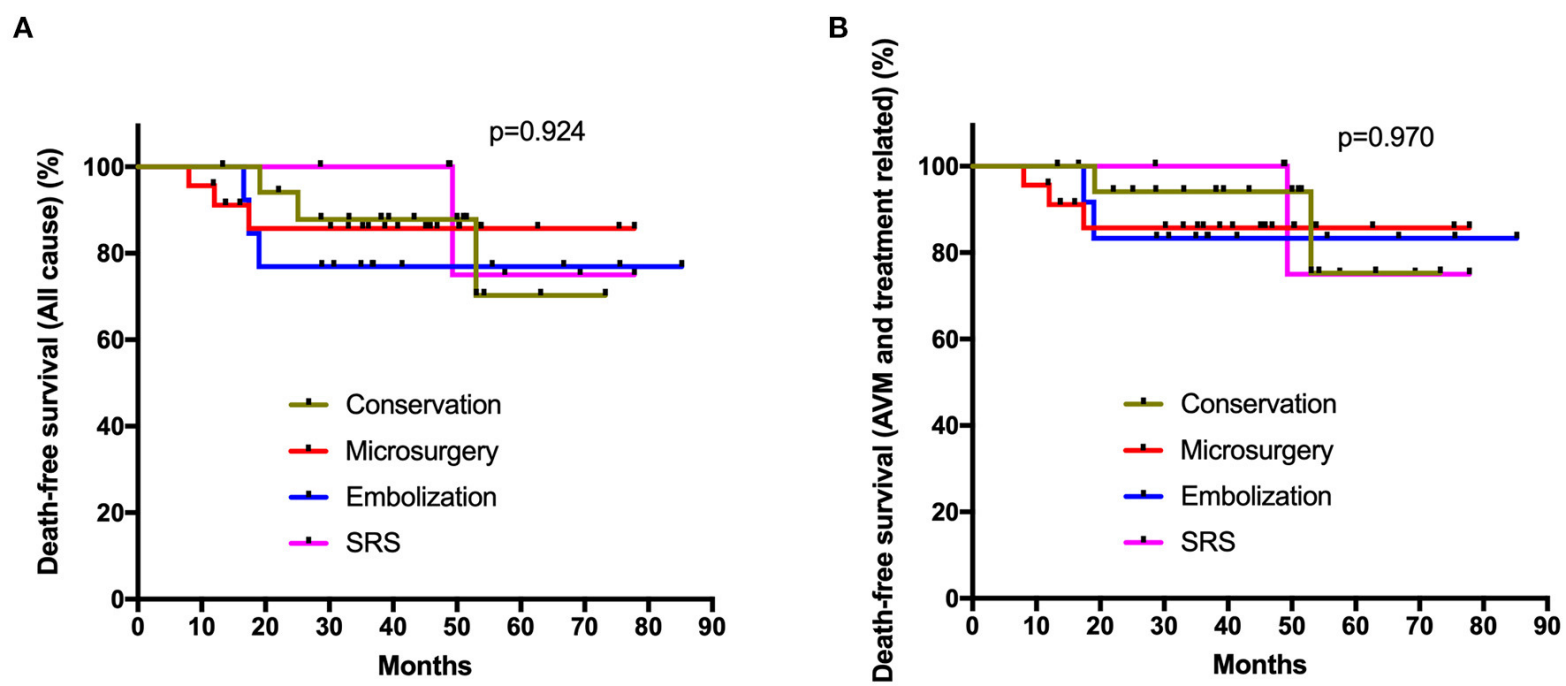
Kaplan-Meier plot. The Kaplan-Meier analysis showed no significant difference in the death-free survival among different management modalities (**A**: all causes, *p* = 0.924; **B**: AVM and treatment related, *p* = 0.970; log-rank test).

All the 71 elderly AVMs were followed up clinically and angiographically for an average of 4.2 ± 2.3 years (Table 2). The microsurgery group had a significantly higher obliteration rate than embolization and SRS groups (*p* < 0.001, *p* = 0.007, respectively). In terms of long-term mRS score, neurological disabilities (mRS > 2), and worsened mRS, there were no significant differences among the four management modalities (*p* = 0.721, *p* = 0.431, *p* = 0.648, respectively). We showed that 68.0% cases in the intervention group and 81.0% in the conservation group could achieve favorable outcomes (mRS ≤ 2). However, the Poisson rate test of annualized rupture risk for conservation and intervention during follow-up was significant (4.4 vs. 0.5%, *p* = 0.040). Twelve patients (16.9%) died during 295.1 patient-years clinical follow-up. The main cause of death in the conservation group was subsequent hemorrhage (3 of 4, 75.0%), while the main cause of death in the intervention group was treatment-related complications (5 of 7, 71.4%). One patient in the conservation group and one in the embolization group died of MACEs. The Kaplan-Meier analysis (Figure 2) showed no significant difference in the death-free survival among different management modalities (all causes, *p* = 0.924; AVM and treatment-related, *p* = 0.970).

**Figure 3:**
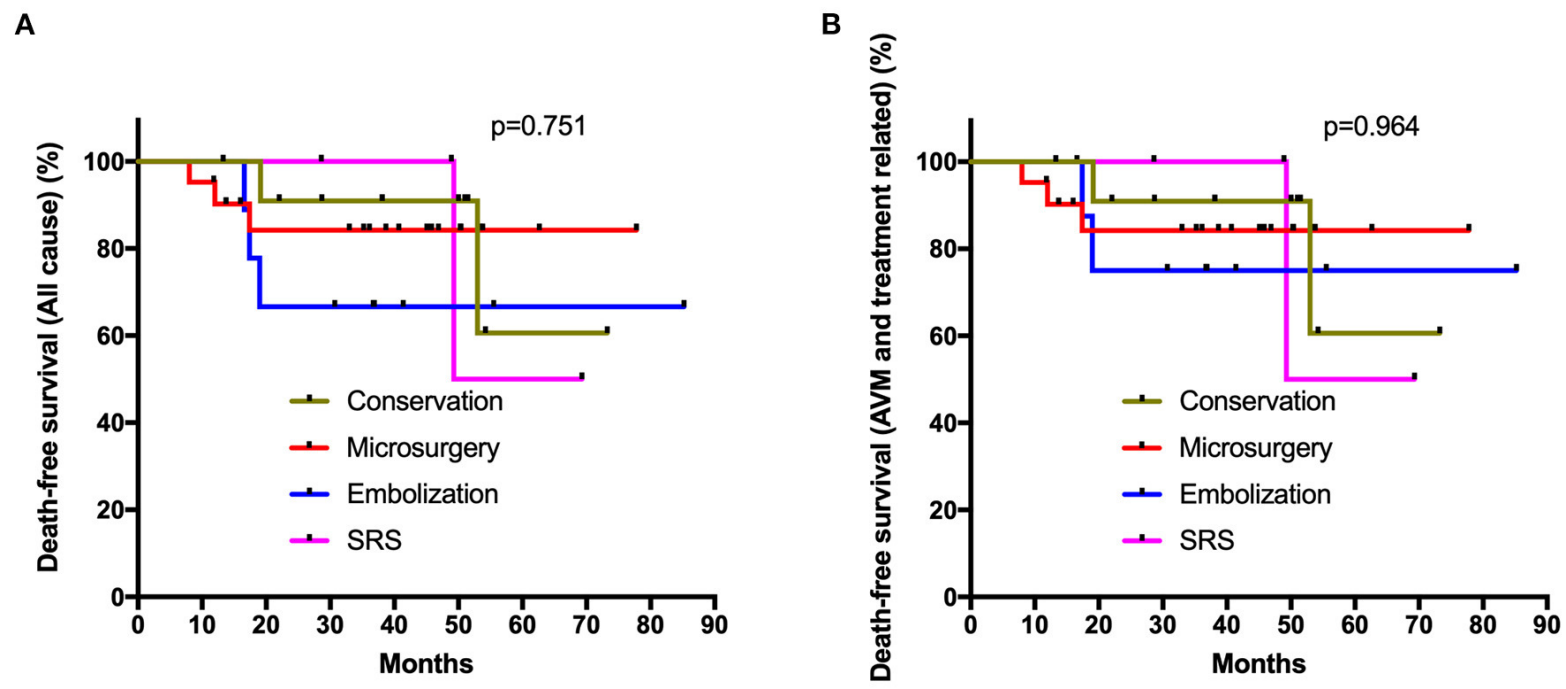
Kaplan-Meier plot. The Kaplan-Meier analysis showed no significant difference in the death-free survival among different management modalities in the ruptured subgroup (**A**: all causes, *p* = 0.751; **B**: AVM and treatment related, *p* = 0.964; log-rank test).

In the subgroup analysis, all prognostic parameters were similar among different management modalities in the unruptured subgroup, except for the obliteration rates. In the ruptured group, the discharge mRS and long-term mRS were similar among these four management modalities after adjusted *post-hoc* Bonferroni correction analysis (*p* = 0.096, *p* = 0.904, respectively) (Table 3). The same was true for >65 years old ruptured AVMs (conservation vs. intervention: *p* = 0.095, *p* = 0.892, respectively; conservation vs. microsurgery: *p* = 0.106, *p* = 0.765, respectively) (Supplementary Material 2). The Poisson rate test of annualized rupture risk for conservation and intervention during follow-up in the ruptured subgroup was significant (*p* = 0.014). However, the Kaplan-Meier analysis (Figure 3) showed no significant difference in the death-free survival among different management modalities in the ruptured subgroup (all causes, *p* = 0.751; AVM and treatment-related, *p* = 0.964).

**Table 3:** Perioperative complications and long-term outcomes among different treatment modalities in the ruptured and unruptured elderly AVMs.

**Characteristics (Ruptured)**	**Conservation (*n* = 13)**	**Microsurgery (*n* = 28)**	**Embolization (*n* = 9)**	**SRS (*n* = 4)**	***p*-value**
Age (years)	63.7 ± 4.2	65.8 ± 3.6	64.2 ± 1.8	62.3 ± 2.2	0.122
Age (>65 years)	5 (38.5)	18 (64.3)	3 (33.3)	1 (25.0)	0.166
Admission mRS score	1.1 ± 0.9	2.1 ± 1.4	1.0 ± 0.5	0.8 ± 0.5	0.010^*^
SM grade (IV-V)	3 (23.1)	5 (17.9)	0 (0.0)	0 (0.0)	0.173
Perioperative complications	NA	18 (64.3)	2 (22.2)	0 (0.0)	0.005^*^
Intracranial hemorrhage	NA	4 (14.3)	1 (11.1)	0 (0.0)	0.560
Epilepsy	NA	1 (3.6)	0 (0.0)	0 (0.0)	0.679
New-onset neurofunctional deficit	NA	6 (21.4)	2 (22.2)	0 (0.0)	0.398
Wound infection	NA	2 (7.1)	0 (0.0)	0 (0.0)	0.456
Intracranial infection	NA	7 (25.0)	0 (0.0)	0 (0.0)	0.050
Lung infection	NA	5 (17.9)	2 (22.2)	0 (0.0)	0.435
MACEs	NA	2 (7.1)	1 (11.1)	0 (0.0)	0.678
DVT	NA	6 (21.4)	2 (22.2)	0 (0.0)	0.398
Electrolyte disturbance	NA	7 (25.0)	2 (22.2)	0 (0.0)	0.345
Discharge mRS	0.8 ± 0.7	1.8 ± 1.5	1.9 ± 1.8	0.8 ± 0.5	0.096
Follow-up duration (years)	4.0 ± 2.1	4.3 ± 2.7	3.1 ± 1.8	4.1 ± 1.4	0.660
Obliteration	0 (0.0)	25 (89.3)	1 (11.1)	2 (50.0)	<0.001^*^
Long-term mRS score	1.9 ± 2.4	2.2 ± 2.2	2.4 ± 2.7	1.5 ± 3.0	0.904
Neurological disability (mRS > 2)	3 (23.1)	11 (39.3)	3 (33.3)	1 (25.0)	0.748
Worsened mRS	4 (30.8)	7 (25.0)	3 (33.3)	1 (25.0)	0.957
No. of subsequent hemorrhage	4	1	0	0	0.034^*^
Annualized rupture risk^a^	7.8%	0.8%	0.0%	0.0%	
Death	3 (23.1)	4 (14.3)	3 (33.3)	1 (25.0)	0.654
Annualized mortality (all causes)	5.8%	3.3%	10.6%	6.1%	
Annualized mortality (AVM-related)	5.8%	0.8%	0.0%	6.1%	
Annualized mortality (treatment-related)	NA	2.5%	7.1%	0.0%	
Annualized mortality (other causes)	0.0%	0.0%	3.5%	0.0%	
**Characteristics (Unruptured)**	**Conservation (*****n*** **= 8)**	**Microsurgery (*****n*** **= 2)**	**Embolization (*****n*** **= 4)**	**SRS (*****n*** **= 3)**	***p*****-value**
Age (years)	65.7 ± 3.5	65.8 ± 1.0	64.0 ± 3.4	62.0 ± 2.0	0.380
Age (>65 years)	4 (50.0)	1 (50.0)	2 (50.0)	0 (0.0)	0.305
Admission mRS score	1.1 ± 1.0	1.0 ± 0.0	1.3 ± 1.0	0.3 ± 0.6	0.558
SM grade (IV-V)	0 (0.0)	0 (0.0)	1 (25.0)	0(0.0)	0.375
Perioperative complications	NA	0 (0.0)	1 (25.0)	0 (0.0)	0.411
Intracranial hemorrhage	NA	0 (0.0)	1 (25.0)	0 (0.0)	0.411
Epilepsy	NA	0 (0.0)	0 (0.0)	0 (0.0)	>0.999
New-onset neurofunctional deficit	NA	0 (0.0)	1 (25.0)	0 (0.0)	0.411
Wound infection	NA	0 (0.0)	0 (0.0)	0 (0.0)	>0.999
Intracranial infection	NA	0 (0.0)	0 (0.0)	0 (0.0)	>0.999
Lung infection	NA	0 (0.0)	0 (0.0)	0 (0.0)	>0.999
MACEs	NA	0 (0.0)	0 (0.0)	0 (0.0)	>0.999
DVT	NA	0 (0.0)	0 (0.0)	0 (0.0)	>0.999
Electrolyte disturbance	NA	0 (0.0)	0 (0.0)	0 (0.0)	>0.999
Discharge mRS	1.0 ± 0.8	1.0 ± 0.0	1.3 ± 1.3	0.3 ± 0.6	0.568
Follow-up duration (years)	4.8 ± 2.5	4.4 ± 2.7	4.3 ± 1.9	5.1 ± 1.2	0.960
Obliteration	0 (0.0)	2 (100.0)	0 (0.0)	2 (66.7)	0.002^*^
Long-term mRS score	1.3 ± 2.1	0.0 ± 0.0	1.5 ± 1.0	0.7 ± 1.2	0.728
Neurological disability (mRS > 2)	1 (12.5)	0 (0.0)	1 (25.0)	0 (0.0)	0.618
Worsened mRS	0 (0.0)	0 (0.0)	2 (50.0)	1 (33.3)	0.090
No. of subsequent hemorrhage	0	0	0	0	>0.999
Annualized rupture risk	0.0%	0.0%	0.0%	0.0%	
Death	1 (12.5)	0 (0.0)	0 (0.0)	0 (0.0)	0.664
Annualized mortality (all causes)	2.6%	0.0%	0.0%	0.0%	
Annualized mortality (AVM-related)	0.0%	0.0%	0.0%	0.0%	
Annualized mortality (treatment-related)	NA	0.0%	0.0%	0.0%	
Annualized mortality (other causes)	2.6%	0.0%	0.0%	0.0%	

*AVM, Arteriovenous Malformation; DVT, Deep Vein Thrombosis; MACE, Major Adverse Cardiac Events; mRS, modified Rankin Scale; SM grade, Spetzler-Martin grade; SRS, Stereotactic Radiosurgery*.

*Values are expressed as number of cases (%) or mean ± standard deviation, unless otherwise indicated*.

a *Poisson rate test of annualized rupture risk for conservation and intervention during follow-up in the ruptured subgroup is significant (p = 0.014)*.

* *Statistical significance (p < 0.05)*.

### Predictors of Long-Term Unfavorable Outcomes (mRS > 2)

During the clinical follow-up, 20 patients experienced long-term unfavorable outcomes (mRS > 2), including four (20.0%) in the conservation group, 11 (55.0%) in the microsurgery group, four (20.0%) in the embolization group, and one (5.0%) in the SRS group (Table 4). In the univariable regression analysis, age (>65 years) (75.0 vs. 37.3%, *p* = 0.006), higher admission mRS score (2.2 ± 1.4 vs. 1.2 ± 1.0, *p* = 0.003), and SM grade IV-V (30.0 vs. 7.8%, *p* = 0.024) were associated with long-term unfavorable outcomes (mRS > 2). In the multivariate logistic regression analysis, age (>65 years) (OR 4.276, 95% CI 1.155–15.839, *p* = 0.030), higher admission mRS score (OR 1.749, 95% CI 1.048–2.920, *p* = 0.033), and SM grade IV-V (OR 6.079, 95% CI 1.182–31.258, *p* = 0.031) were significantly associated with long-term unfavorable outcomes (mRS > 2) in the whole cohort. The management modalities and complete obliteration rate had no significant correlation with long-term unfavorable outcomes (mRS > 2) (*p* = 0.431, *p* = 0.951, respectively). In the intervention group, higher admission mRS score (OR 3.070, 95% CI 1.559–6.043, *p* = 0.001) and complete obliteration (OR 0.146, 95% CI 0.026–0.828, *p* = 0.030) were the independent predictors of long-term unfavorable outcomes (mRS > 2). In the microsurgical resection group, higher admission mRS score (OR 4.010, 95% CI 1.321–12.175, *p* = 0.014) and SM grade IV-V (OR 39.048, 95% CI 1.016–1500.618, *p* = 0.049) were the independent predictors of long-term unfavorable outcomes (mRS > 2).

**Table 4:** Univariate and multivariate logistic regression analysis for long-term unfavorable outcomes (mRS > 2).

**Characteristics (Total)**	**Univariate**			***p*-value**	**Multivariate**	***p*-value**
	**Present (*n* = 20)**	**Absent (*n* = 51)**	**OR (95% CI)**		**OR (95% CI)**	
Sex (male)	15 (75.0)	37 (72.5)	0.881 (0.270–2.879)	0.834		
Age (>65 years)	15 (75.0)	19 (37.3)	5.053 (1.583–16.125)	0.006^*^	4.276 (1.155–15.839)	0.030^*^
Hemorrhagic presentation	18 (90.0)	36 (70.6)	3.750 (0.772–18.209)	0.085		
Admission mRS score	2.2 ± 1.4	1.2 ± 1.0	2.001 (1.260–3.179)	0.003^*^	1.749 (1.048–2.920)	0.033^*^
Supratentorial location	14 (70.0)	37 (72.5)	0.883 (0.283–2.752)	0.830		
Size (cm)	3.3 ± 1.7	2.8 ± 1.4	1.024 (0.990–1.059)	0.168		
SM grade (IV–V)	6 (30.0)	4 (7.8)	5.036 (1.243–20.397)	0.024^*^	6.079 (1.182–31.258)	0.031^*^
Management modalities				0.468		
Conservation	4 (20.0)	17 (33.3)	Ref.			
Microsurgery	11 (55.0)	19 (37.3)	0.768 (0.191–3.089)	0.710		
Embolization	4 (20.0)	9 (17.6)	0.288 (0.031–2.714)	0.277		
SRS	1 (5.0)	6 (11.8)	0.406 (0.109–1.519)	0.181		
Complete obliteration	8 (40.0)	20 (39.2)	1.033 (0.359–2.972)	0.951		
**Characteristics (intervention)**	**Univariate**			***p*****-value**	**Multivariate**	***p*****-value**
	**Present (*****n*** **= 16)**	**Absent (*****n*** **= 34)**	**OR (95% CI)**		**OR (95% CI)**	
Sex (male)	12 (75.0)	27 (79.4)	1.286 (0.316–5.235)	0.726		
Age (>65 years)	12 (75.0)	13 (38.2)	4.846 (1.287–18.255)	0.020^*^		
Hemorrhagic presentation	15 (93.8)	26 (76.5)	4.615 (0.525–40.577)	0.168		
Admission mRS score	2.4 ± 1.5	1.2 ± 1.0	2.279 (1.319–3.939)	0.003^*^	3.070 (1.559–6.043)	0.001^*^
Supratentorial location	11 (68.8)	25 (73.5)	0.792 (0.215–2.915)	0.726		
Size (cm)	3.3 ± 1.7	2.7 ± 1.4	1.024 (0.985–1.064)	0.232		
SM grade (IV-V)	4 (25.0)	2 (5.9)	5.333 (0.862–32.997)	0.072		
Management modalities				0.544		
Microsurgery	11 (68.8)	19 (55.9)	Ref.			
Embolization	4 (25.0)	9 (26.5)	0.768 (0.191–3.089)	0.710		
SRS	1 (6.3)	6 (17.6)	0.288 (0.031–2.714)	0.277		
Complete obliteration	8 (50.0)	20 (58.8)	0.578 (0.177–1.882)	0.363	0.146 (0.026–0.828)	0.030^*^
**Characteristics (Microsurgery)**	**Univariate**			***p*****-value**	**Multivariate**	***p*****-value**
	**Present (*****n*** **= 11)**	**Absent (*****n*** **= 19)**	**OR (95% CI)**		**OR (95% CI)**	
Sex (male)	8 (72.7)	14 (73.7)	1.050 (0.197–5.602)	0.954		
Age (>65 years)	9 (81.8)	10 (52.6)	4.050 (0.685–23.949)	0.123		
Hemorrhagic presentation	11 (100)	17 (89.5)	1.0^*^10^9^ (0.000-)	>0.999		
Admission mRS score	3.1 ± 1.3	1.4 ± 1.1	2.775 (1.377–5.595)	0.004^*^	4.010 (1.321–12.175)	0.014^*^
Supratentorial location	9 (81.8)	15 (78.9)	1.200 (0.182–7.926)	0.850		
Size (cm)	3.7 ± 1.7	3.0 ± 1.2	1.041 (0.985–1.099)	0.156		
SM grade (IV-V)	4 (36.4)	1 (5.3)	15.000 (1.449–155.313)	0.023^*^	39.048 (1.016–1500.618)	0.049^*^
Complete obliteration	8 (72.7)	19 (100.0)	0.000 (0.000-)	>0.999		

*CI, Confidence Intervals; mRS, modified Rankin Scale; OR, Odds Ratio; SM grade, Spetzler-Martin grade; SRS, Stereotactic Radiosurgery*.

*Values are expressed as number of cases (%) or mean ± standard deviation, unless otherwise indicated*.

* *Statistical significance (p < 0.05)*.

## Discussion

As the life expectancy of the overall population continues to increase in this global aging society, whether radical interventions for elderly AVMs can achieve longer survival time and better neurological functional state than conservative management is an urgent problem to be solved (Harbaugh and Harbaugh, 1994; Lanzino et al., 1997; Hashimoto et al., 2004; Nagata et al., 2006; Tong et al., 2015; Burkhardt et al., 2018; Chen et al., 2018). We conducted a multicenter retrospective study involving multiple management modalities (conservation, microsurgery, embolization, SRS) for elderly AVMs. Our study found that elderly AVMs demonstrated an aggressive natural history, with an annualized natural rupture rate of 9.4%. The long-term outcomes and mortality in elderly AVMs were similar among different management modalities in the ruptured and unruptured groups. Although the intervention (microsurgery, embolization, SRS) could significantly reduce the risk of subsequent hemorrhage than conservation in the ruptured subgroup, it should be noted that the main cause of death in the intervention group was treatment-related complications. Therefore, we do not recommend intervention for unruptured elderly AVMs, and for those ruptured, we should carefully weigh the risk of subsequent hemorrhage and treatment-related complications. Besides, uncomplete obliteration was found to be significantly associated with unfavorable outcomes (mRS > 2) in the intervention group. Therefore, complete obliteration should be a must if the intervention strategy were chosen.

### Natural History

About 30 years ago, the elderly AVMs were considered relatively benign, and the risk of bleeding would decrease as the patient reached middle age (Luessenhop and Rosa, 1984; Heros and Tu, 1987; Goldberg et al., 2018). However, in recent decades, this view was challenged by the increased sample size of elderly AVMs due to the aging of population and the refinement of neuroimaging modalities (Crawford et al., 1986; Brown et al., 1996; Hetts et al., 2014; Pabaney et al., 2016; Burkhardt et al., 2018). Kim et al. conducted a multicenter, individual patient-level meta-analysis, and showed that increasing age is an independent predictor of hemorrhage during follow-up (Kim et al., 2014). However, no previous study calculated the annualized rupture rate in elderly AVMs. This study defined the observational duration of natural history as the interval from the first diagnosis to admission. Finally, we calculated an annualized rupture risk of 9.4% in the elderly AVM cohort, which was higher than that in the overall AVM cohort (2–4% per year) published in previous studies (Itoyama et al., 1989; Goldberg et al., 2018). We demonstrated that the elderly AVMs may be even more aggressive than young AVMs, rather than benign lesions as reported.

### Conservation or Intervention

Generally, SM grade I/II/III are amenable to intervention (SM grade I/II: microsurgical resection, endovascular embolization, SRS; SM grade III: multimodal approach), and SM grade IV/V are recommended to be monitored unless ruptured (Derdeyn et al., 2017). In reviewing the previous literature on elderly AVMs, we found no subgroup analysis was performed in terms of rupture and unruptured presentation. In 2014, a randomized trial of unruptured brain AVMs (ARUBA) concluded that medical therapy was superior in preventing stroke and death over a follow-up period of 33 months (Mohr et al., 2014). In this study, the long-term outcomes were similar (long-term mRS score and mortality) among different management modalities in the unruptured subgroup. No patients in the conservation group occurred hemorrhage event during clinical follow-up. Therefore, it may not be advisable to intervene for unruptured elderly AVMs because of the low rupture risk and the relatively shorter life expectancy.

In the ruptured elderly AVMs, the risk of severe complications after intervention must be weighed against the natural re-rupture risk of lesions. Previous studies have drawn ambiguous attitudes about whether to intervene with elderly AVMs. Although the rupture risk is positively correlated with age progression, advanced age is also significantly correlated with a higher risk of neurological disabilities and mortality after the intervention (Ding and Liu, 2013). Hashimoto et al. reported that 69.6% of the elderly AVMs could achieve satisfactory outcomes after microsurgery, so they recommended microsurgical resection for SM grade I-II AVMs (Hashimoto et al., 2004). One recent study conducted by Burkhardt et al. proposed that 71% of elderly AVMs could achieve favorable outcomes after microsurgical resection, and they recommended microsurgical resection for carefully selected patients (Burkhardt et al., 2018). However, in Burkhardt's study, it should be noted that 84% could achieve favorable outcomes after conservation (higher than intervention). Besides, several studies recommended SRS for elderly AVMs because the advanced age does not reduce the obliteration rate or increase the incidence of complications (Ding et al., 2015; Chen et al., 2018; Hasegawa et al., 2018). In this study, the long-term outcomes were similar (long-term mRS score and mortality) among different management modalities in the ruptured subgroup. On the one hand, intervention could significantly reduce the risk of subsequent hemorrhage compared with conservation in the ruptured elderly AVMs. Nevertheless, on the other hand, we also found that the main cause of death in the microsurgery and embolization group was treatment-related complications. Therefore, the management modality selection for ruptured elderly AVMs should be determined after carefully weighing the risk of subsequent hemorrhage and treatment-related complications.

### Predictors of Unfavorable Outcomes (mRS > 2)

Spetzler and Martin proposed the SM grading system to predict the morbidity and mortality of the operative treatment (Spetzler and Martin, 1986). Several previous studies indicated that the SM grading system was also applicable to elderly AVMs (Tong et al., 2015; Burkhardt et al., 2018). In this study, SM grade IV-V and higher admission mRS score were found to be significantly associated with long-term unfavorable outcomes (mRS > 2) both in the whole cohort and microsurgery group, which was consistent with previous studies (Tong et al., 2015). Burkhardt et al. and Nagata et al. suggested that age >65 years was an independent predictor of unfavorable outcomes after microsurgical resection (Nagata et al., 2006; Burkhardt et al., 2018). In our study, we only found a significant correlation between age >65 years and unfavorable outcomes in the whole elderly AVM cohort. In the intervention group, higher admission mRS scores and uncomplete obliteration were correlated with unfavorable outcomes, which means that we must obliterate the lesions completely as we operated.

We acknowledge that our study has several limitations. First, the selection bias exists due to the retrospective nature of our study design. Many elderly AVMs with lower rupture risk may be recommended for conservative treatment without hospitalization, which would increase the number of ruptured patients in the study cohort, and thus render an overestimation of the aggressiveness of their natural history. Second, the sample size was small, especially in the embolization group and SRS group, impeding us from conducting in-depth analysis in each management modality. Third, it may not be appropriate to define the elderly as >60 years old in today's aging population. However, the retirement age is 60 years old in China, and we thought it is reasonable for us to define it as such in this study. Fourth, the follow-up duration is relatively short (4.2 ± 2.3 years). Previous studies have confirmed that 5–10 years after the first rupture may be the peak period of rebleeding. Therefore, the similarity in long-term outcomes may be due to the absence of rebleeding events in the conservation group during our follow-up. Our study shows consistencies and discrepancies compared with previous studies, and further multicenter studies with larger sample sizes are needed to verify our findings.

## Conclusions

The natural history of elderly AVMs is not benign, with an annualized natural rupture rate of 9.4%. The long-term neurological outcomes and mortality of different management modalities for elderly AVMs were similar both in the ruptured and unruptured subgroup. Although intervention could significantly reduce the risk of subsequent hemorrhage than conservation in the ruptured subgroup, the treatment-related complications were the main cause of death in the intervention group. All in all, intervention for unruptured elderly AVMs was not recommended. For the ruptured elderly AVMs, we should carefully weigh the risk of subsequent hemorrhage and treatment-related complications before formulating individualized treatment strategies. Besides, complete obliteration is required if we chose to intervene.

## Data Availability Statement

The raw data supporting the conclusions of this article will be made available by the authors, without undue reservation.

## Ethics Statement

The studies involving human participants were reviewed and approved by the ethics committee of Beijing Tiantan Hospital and Peking University International Hospital. The patients/participants provided their written informed consent to participate in this study.

## Author Contributions

YC conceived the idea, designed the paper, and wrote the manuscript. YC, DY, and LM performed the statistical analysis. ZL, YC, DY, LM, and YaZ collected the data. LM, XC, HW, and YuZ funded the study. HW, XY, HJ, YL, DG, SS, AL, SW, XC, and YuZ critically revised the manuscript and approved the final manuscript as submitted. All authors agreed to be accountable for all aspects of the work in ensuring that questions related to the accuracy or integrity of any part of the work are appropriately investigated and resolved.

## Conflict of Interest

The authors declare that the research was conducted in the absence of any commercial or financial relationships that could be construed as a potential conflict of interest.
