# Supplementary material for: Long-Term Outcomes of Elderly Brain Arteriovenous Malformations After Different Management Modalities: A Multicenter Retrospective Study
Source: Front Aging Neurosci. 2021 Feb 18;13:609588. doi: 10.3389/fnagi.2021.609588 (PMC7930621; doi:10.3389/fnagi.2021.609588)
Supplement: Supplementary file 1 [file Data_Sheet_1.PDF]

Supplementary Material 1. Comparison between follow-up and lost follow-up elderly AVMs.

Characteristics	Follow up (n=71)	Lost follow-up (n=17)	p value
Sex (male)	52 (73.2)	15 (88.2)	0.324
Age (years)	64.7±3.5	65.1±2.6	0.700
Age (>65 years)	34 (47.9)	7 (41.2)	0.618
Onset manifestation (primary)			
Hemorrhage	54 (76.1)	15 (88.2)	0.442
Seizure	4 (5.6)	1 (5.9)	>0.999
Neurofunctional deficit	4 (5.6)	1 (5.9)	>0.999
Others	9 (12.7)	0 (0.0)	0.121
No. of hemorrhagic events between diagnosis and treatment	14	3	
Annualized rupture risk	9.4%	13.8%	0.551
Admission mRS score	1.4±1.3	1.1±1.1	0.698
Size (cm)	2.9±1.5	2.8±1.7	0.975
Eloquent area	41 (57.7)	8 (47.1)	0.426
Supratentorial location	51 (71.8)	12 (70.6)	>0.999
Deep venous drainage	31 (43.7)	4 (23.5)	0.128
SM grade			0.334
I	15 (21.1)	6 (35.3)	
II	22 (31.0)	7 (41.2)	
III	24 (33.8)	2 (11.8)	
IV	7 (9.9)	1 (5.9)	
V	3 (4.2)	1 (5.9)	
Treatment modalities			0.419
Conservation	21 (29.6)	2 (11.8)	
Microsurgery	30 (42.3)	10 (58.8)	
Embolization	13 (18.3)	3 (17.6)	
SRS	7 (9.9)	2 (11.8)	
Follow-up duration (years)	4.2±2.3	3.7±0.5	0.826
Angioarchitecture characteristics (DSA available, n=53)	n=53	n=11	
Drainage venous stenosis	27 (50.9)	5 (45.5)	0.740
Long venous drainage	32 (60.4)	6 (54.5)	0.746
Deep perforating arteries	17 (32.1)	4 (36.4)	>0.999
Diffuse nidus	21 (39.6)	4 (36.4)	>0.999
Aneurysms (flow-related)	11 (20.8)	2 (18.2)	>0.999
Discharge mRS	1.3±1.3	0.9±1.0	0.244

AVM= Arteriovenous Malformation; DSA= Digital Subtraction Angiography; mRS= modified Rankin Scale; SM grade= Spetzler-Martin grade; SRS= Stereotactic Radiosurgery

Values are expressed as number of cases (%) or mean ± standard deviation, unless otherwise indicated.

Supplementary Material 2. Prognostic comparison between conservation and intervention in >65 years old ruptured AVMs.

Characteristics (Ruptured)	Conservation (n=5)	Intervention (n=22)	p value	Microsurgery (n=18)	p value
Perioperative complications	NA	13 (59.1)		12	
Intracranial hemorrhage	NA	4 (18.2)		3	
Epilepsy	NA	1 (4.5)		1	
New-onset neurofunctional deficit	NA	7 (31.8)		6	
Wound infection	NA	1 (4.5)		1	
Intracranial infection	NA	5 (22.7)		5	
Lung infection	NA	5 (22.7)		4	
MACEs	NA	2 (9.1)		1	
DVT	NA	4 (18.2)		4	
Electrolyte disturbance	NA	5 (22.7)		4	
Discharge mRS	0.8±0.8	2.0±1.5	0.095	1.9±1.4	0.106
Follow-up duration (years)	3.1±2.4	4.2±2.5	0.373	4.7±2.5	0.211
Obliteration	0 (0.0)	15 (68.2)	0.010*	15	0.002*
Long-term mRS score	2.8±2.9	2.6±2.3	0.892	2.4±2.1	0.765
Neurological disability (mRS>2)	2 (40.0)	10 (45.5)	>0.999	8	>0.999
Worsened mRS	2 (40.0)	8 (36.4)	>0.999	6	>0.999
No. of subsequent hemorrhage	2 (40.0)	2 (9.1)	0.144	1	>0.107
Annualized rupture risk&	13.0%	2.2%	0.111	1.2%	0.067
Death	2 (40.0)	4 (18.2)	0.303	2	0.194
Annualized mortality (all causes)	13.0%	4.3%	0.248	2.4%	0.127
Annualized mortality (AVM-related)	13.0%	1.1%	0.058	1.2%	0.067
Annualized mortality (treatment-related)	NA	2.2%		1.2%	
Annualized mortality (other causes)	0.0%	1.1%	0.857	0.0%	>0.999

AVM= Arteriovenous Malformation; DVT= Deep Vein Thrombosis; MACE= Major Adverse Cardiac Events; mRS= modified Rankin Scale; SM grade= Spetzler-Martin grade; SRS= Stereotactic Radiosurgery

Values are expressed as number of cases (%) or mean ± standard deviation, unless otherwise indicated.

*Statistical significance (p<0.05)
